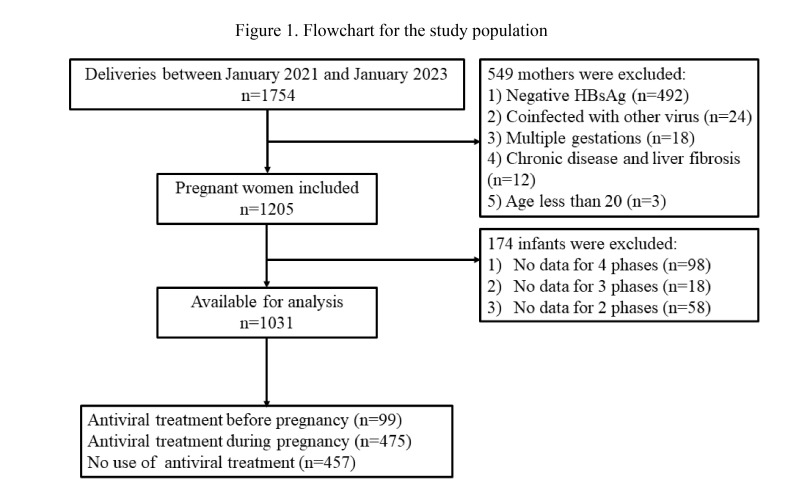# Maternal and infant growth outcomes following preconception antiviral therapy in chronic hepatitis B virus infection: A retrospective cohort study

**DOI:** 10.1097/MD.0000000000049131

**Published:** 2026-06-12

**Authors:** Xing-Ran Tao, Shi-Jing Gao, Jia Jia, Guorong Han

**Affiliations:** a Department of Obstetrics and Gynecology, The Second Hospital of Nanjing, and Affiliated to Nanjing University of Chinese Medicine, Nanjing, Jiangsu Province, China.

**Keywords:** growth parameter, hepatitis B virus, nucleos(t)ide analogue, preconception, pregnancy outcome

## Abstract

Despite the clinical significance, data are limited on pregnancy outcomes and infant growth among women with chronic hepatitis B virus (HBV) infection treated with antiviral treatment before pregnancy (ATBP). This study aimed to evaluate the effects of ATBP on maternal pregnancy outcomes and infant growth parameters. A retrospective cohort study was conducted among 1031 women aged ≥20 years with singleton pregnancies and chronic HBV infection, recruited from January 2021 to January 2023. Propensity score matching was applied to balance baseline characteristics across 3 groups: 99 women receiving ATBP, 99 receiving antiviral treatment during pregnancy (ATDP), and 99 receiving no antiviral treatment. Pregnancy outcomes were analyzed using modified Poisson regression, while infant growth parameters were assessed by generalized linear regression, stratified by sex. After matching, mothers in the ATBP group exhibited a significantly lower risk of gestational abnormal alanine aminotransferase compared to those in the ATDP group. No significant differences were observed in maternal pregnancy outcomes (such as hypertensive disorders of pregnancy, gestational diabetes mellitus, and preterm birth) and in the growth parameters of their infants in the ATBP group versus the other 2 groups. Only 1 infant in this study, who was in the ATDP group and received antiviral treatment starting at 24 weeks of gestation, was diagnosed with chronic HBV infection. Our findings indicated that ATBP was associated with a lower risk of gestational alanine aminotransferase abnormalities in women with chronic HBV infection. However, we did not find a difference in maternal pregnancy outcomes or infant growth in the first year of life. These results provide insights for clinicians managing pregnancy in this population.

## 1. Introduction

Hepatitis B virus (HBV) infection poses a significant threat to global public health, with a prevalence of 3.8% globally and 5.6% in China.^[1,2]^ Individuals with chronic HBV infection are prone to experience liver complications.^[3]^ Clinical practice guidelines underscore the indication for antiviral therapy in chronic HBV infection among individuals with specific risk factors. These factors include a family history of liver cirrhosis or hepatocellular carcinoma, an age exceeding 30 years, the presence of significant hepatic inflammation or fibrosis, and HBV-related extrahepatic manifestations.^[4,5]^ In addition, the concept of expanding treatment has led to increased utilization of nucleos(t)ide analogues (NAs) among women.

Upon the planning or confirming of pregnancy, continuation of treatment is generally advised for women with chronic HBV infection receiving NAs, choosing drugs in the first-line recommendation (telbivudine [LdT], tenofovir disoproxil fumarate [TDF], and tenofovir alafenamide fumarate [TAF]), which are safe in intrauterine exposure.^[6,7]^ During pregnancy, cell-mediated immunity is physiologically modulated to maintain maternal–fetal immune tolerance and prevent rejection of the semi-allogeneic fetus.^[8,9]^ However, this pregnancy-related immunomodulation may also weaken host immune control of HBV, thereby facilitating viral replication and contributing to hepatic inflammation and elevated alanine aminotransferase (ALT) levels, a sensitive biomarker of hepatic injury.^[10]^ The primary objective of antiviral therapy is to treat active hepatitis and reduce the risk of mother-to-child transmission, which may affect ALT levels. Despite an increasing trend of women initiating antiviral therapy, there is still a dearth of research exploring the impact of NA therapy throughout the entire pregnancy on mothers and their fetuses. We aimed to address this knowledge gap by evaluating the safety of antiviral therapy throughout pregnancy and its impact on the development of infants born to mothers with chronic HBV infection.

## 2. Methods

### 2.1. Study design and study population

This retrospective observational cohort study, conducted among patients who delivered between January 2021 and January 2023 and their infants at The Second Hospital of Nanjing (affiliated with Nanjing University of Chinese Medicine), was performed in accordance with the ethical standards of the World Medical Association Declaration of Helsinki. The study protocol was reviewed and approved by the Clinical Ethics Committee of The Second Hospital of Nanjing (approval number: 2023-LS-ky-036). The requirement for written informed consent was waived by the committee due to the retrospective design and the use of fully anonymized patient data.

All pregnant women with chronic HBV infection received the standard of care. They underwent many screenings, including *Treponema pallidum*, hepatitis viruses (HAV, HBV, HCV, HDV, and HEV), human immunodeficiency virus (HIV), and liver function during their initial prenatal visit. Upon detection of hepatitis B surface antigen (HBsAg) positivity, HBV markers, including hepatitis B e antigen (HBeAg) and quantitative HBV DNA, were assessed, and the management plan was meticulously documented. Patients underwent regular monitoring of liver function and HBV DNA levels every 1 to 2 months throughout gestation. In the context of pregnancy and HBV management, antiviral therapy intervention was initiated during 24 to 28 weeks of gestation, contingent upon maternal viral loads exceeding 5.3 log_10_ IU/mL.^[11]^ Antiviral therapy was administered according to hospital policy and national guidelines. The NAs used included LdT (600 mg once daily), TDF (300 mg once daily), and TAF (25 mg once daily). With the patient’s informed consent and in accordance with the severity of their condition, prompt administration of NA was warranted if ALT (>40 U/L) arose during pregnancy and ruled out other causes.

Hepatitis B vaccine (10 µg/0.5 mL) and 100 IU hepatitis B immune globulin should be given within 12 hours of the birth of the child, with 2 more doses (10 µg/0.5 mL) planned at 1 and 6 months. This was the protocol for managing newborns born to women who had chronic HBV infection. Postvaccination serologic testing was recommended at least 1 month following the final dose of the vaccine.

The study cohort included women aged 20 or older with singleton pregnancies, hospital deliveries, and diagnosed with chronic HBV infection, defined as documented HBsAg positivity for at least 6 months prior to pregnancy (confirmed by at least 2 separate tests). Exclusion criteria included a new diagnosis of HBV in this pregnancy, and HBsAg disappearing in 6 months; discontinued NA drug after antiviral therapy; coinfection with other viruses (*Treponema pallidum*, HIV, HAV, HCV, HDV, and HEV); a history of mental illness; comorbidities (diabetes mellitus, hypertension); tumor presence; or liver fibrosis. The condition of patients with mental illness might impair their adherence to treatment. Coinfection with other viruses, diabetes mellitus, and hypertension were complex pathological conditions that had an impact on the response to treatment and outcome measures. In addition, the treatment might affect liver function. However, those with a history of hypertensive disorders of pregnancy (HDP) or gestational diabetes mellitus (GDM) were included in this study.

For the follow-up data of children, 174 exhibited multiple absences across the 4 follow-up phases (1, 3, 6, and 12 months). Ultimately, our analysis encompassed 1031 mothers and their offspring (Fig. S1, Supplemental Digital Content). Based on common experience in clinical long-term follow-up projects, these losses to follow-up were generally attributable to non-study-specific sociodemographic factors, such as changes in contact information (e.g., incorrect or inactive phone numbers), relocation out of the region, or decreased engagement with routine health follow-ups. Participants were stratified into 3 groups based on antiviral treatment status: antiviral treatment before pregnancy (ATBP, n = 99), antiviral treatment during pregnancy (ATDP, n = 475), and no antiviral treatment (NAT, n = 457). In the ATBP group, all women continued the same antiviral regimen throughout pregnancy without interruption. No patient in this group discontinued therapy before or during pregnancy.

### 2.2. Data sources

We extracted clinical and follow-up data from the Hospital Information System and the administrative database of the Maternal and Child Health Information System. For pregnant women diagnosed with chronic HBV infection, the Hospital Information System captured baseline demographics, liver function indices, HBV biomarkers, and detailed delivery records. To ensure patient privacy, we employed encrypted, unique identifiers to facilitate deterministic linkage across administrative databases. Subsequently, we merged data from the Maternal and Child Health Information System, encompassing birth details and infant follow-up. Prior to integration, the administrative database was validated to confirm maternal characteristics, HBsAg status, and delivery information.

### 2.3. Variables

We gathered demographic and clinical characteristics of mothers and infants. Maternal markers included age, body mass index (BMI), fibrosis-4 (FIB-4), gravida, parity, history of cesarean section, positive HBeAg, HBV DNA, antiviral drug, type of delivery, and gestational age. Postpartum indicators of newborns included sex, Apgar score at 1 minute, birth weight, and height of infants. The developmental parameters of infants – the weight, height, and feeding method – were assessed at 1, 3, and 6 months postpartum. The weight, height, teething, and fontanel closure were collected at 12 months postpartum.

Chronic HBV infection was defined as persistent HBsAg positivity for at least 6 months.^[4,6,12,13]^ During the diagnostic process, patients underwent repeated testing for serological HBV. The BMI before pregnancy, calculated as weight (kg) divided by the square of height (m^2^), categorized individuals into distinct weight groups: underweight (BMI < 18.5), normal weight (18.5–23.9), overweight (24–27.9), and obese (BMI > 28).^[14]^ FIB-4 was employed as a noninvasive tool to evaluate liver fibrosis, with a cutoff of <1.45 for predicting no or minimal fibrosis and >3.25 for predicting significant fibrosis.^[15]^ The formula is: FIB-4 = age (years) × AST (U/L)/(platelet count [10^9^/L] × √ALT [U/L]).^[16]^ Cesarean history was the term used to describe a woman’s prior cesarean section experience. Type of delivery refers to the delivery method of pregnancy, whether natural delivery or a cesarean section.

### 2.4. Outcomes

The primary outcome of this study was the risk of abnormal ALT during gestation. Abnormal ALT was defined as having at least 1 measurement >40 U/L (the upper limit of normal) at any time point during pregnancy. To capture this outcome, liver function was monitored per standard clinical protocol every 1 to 2 months during gestation. ALT level was an important index to evaluate the state of the liver, which usually indicated liver injury or inflammation. In addition, elevated ALT levels were often used to guide clinical decisions, such as the need for antiviral therapy. We acknowledge that in this retrospective study, actual monitoring intervals could vary individually; additionally, we also recognized that ALT levels could be affected by many factors, and therefore, there was a risk of confusion. To reduce the risk, we performed propensity score matching for maternal characteristics across groups and used multivariable regression analysis to adjust for possible confounders.

The secondary outcomes were adverse pregnancy outcomes, including HDP, GDM, preterm birth, postpartum hemorrhage, premature rupture of the membranes (PROM), abnormal amniotic fluid, and intrahepatic cholestasis of pregnancy (ICP). A separate assessment helped to clarify the connection between the duration of antiviral therapy and adverse pregnancy outcomes. HDP was defined as gestational hypertension, preeclampsia, eclampsia, or hemolysis, elevated liver enzymes, low platelets (HELLP) syndrome, diagnosed according to the American College of Obstetricians and Gynecologists guidelines. GDM was identified in accordance with the American Diabetes Association’s 2019 recommendations for the diagnosis and treatment of diabetes in pregnancy, using a 75 g oral glucose tolerance test at 24 to 28 weeks of gestation; GDM was diagnosed in those with fasting blood glucose levels ≥5.1 mmol/L, 1-hour plasma glucose levels ≥10.0 mmol/L, or 2-hour plasma glucose levels ≥8.5 mmol/L. Preterm birth was defined as delivery before 37 completed weeks of gestation. Postpartum hemorrhage was defined as blood loss ≥500 mL for vaginal delivery or ≥1000 mL for cesarean section within 24 hours postpartum. PROM was defined as the spontaneous rupture of amniotic membranes before the onset of labor. Abnormal amniotic fluid was diagnosed as oligohydramnios (amniotic fluid index ≤ 5 cm) or polyhydramnios (amniotic fluid index ≥ 25 cm). ICP was defined as a pregnancy-specific disorder occurring in the second and third trimesters, featuring pruritus and elevated serum total bile acids, with postpartum symptom resolution; a fasting serum total bile acid level exceeding 10 μmol/L was used as the diagnostic threshold.

Another secondary outcome was the development of offspring postpartum, specifically focusing on weight, height, feeding methods, tooth eruption, and anterior fontanel closure. These postpartum measurements were selected because they provide crucial insights into the infants’ growth, nutrition, dental development, and general health. By including these parameters, we comprehensively assessed the postnatal well-being of the offspring in our study.

### 2.5. Study size

In this retrospective cohort study, we compared the group of ATBP versus ATDP, with a primary outcome focused on the incidence of abnormal ALT during gestation. The anticipated incidence rates were set at p1 = 4.6% (0%–4.6%) for the ATBP group and p2 = 16.98% (16.98%–25.64%) for the ATDP group.^[17–19]^ We determined the sample size under a two-sided significance level of α = 0.05, aiming for a power of 1 − β = 0.8, and assuming a 1:1 allocation ratio between the 2 groups. These calculations yielded a maximum of 95 participants per group, totaling up to 285 participants for the entire study.

### 2.6. Statistical analysis

Continuous variables with a normal distribution were reported as mean ± standard deviation (SD), analyzed by a two-sample Student *t* test in 2 groups and an analysis of variance in more than 2 groups. Non-normally distributed continuous variables were reported as median (interquartile range [IQR]), analyzed by the Mann–Whitney *U* test in 2 groups and the Kruskal–Wallis test in more than 2 groups. Categorical data, expressed as counts or percentages, were assessed with the Fisher exact test in 2 groups and the chi-square test in more than 2 groups. Baseline maternal characteristics were comparable between the available and absent data groups (Table S1, Supplemental Digital Content). Missing values, <20% of the total, were imputed using the mean imputation method. Subsequently, a propensity score-matched approach with a tolerance of 0.02 was applied to balance the baseline and facilitate 1:1 matching between patients in the ATDP and NAT groups with those in the ATBP group.

Pregnancy outcomes were assessed postpartum, and the growth parameters of children were measured from birth to their first year of life. *Z* scores for height-for-age (HAZ) and weight-for-age (WAZ), adjusted for age and sex according to WHO growth criteria,^[20]^ were computed and transformed using Ln (*Z* score − min + 1) for analysis. The use of age- and sex-specific *Z* scores (WAZ, HAZ) was to eliminate the confounding effects of age and sex on growth indicators. The Ln (*Z* score − min + 1) transformation was applied prior to regression analysis to make the distribution of the data better meet the normality assumption of the models. The impact of NA therapy on pregnancy outcomes was evaluated using relative risk with a 95% confidence interval (CI). Modified Poisson regression was utilized for multivariate analysis, while generalized linear regression was employed to assess the correlation between maternal treatment and children’s growth indicators. Prior to multivariate analyses, all continuous variables were checked for collinearity using the variance inflation factor. The modeling process encompassed a crude model and an adjusted model (considering birth weight, height, and feeding method at 1, 3, and 6 months; and birth weight and height only at 12 months), and stratified analysis for sex-specific differences. It should be noted that the primary comparisons of interest in all the aforementioned analyses were between the ATBP group and each of the other 2 groups (ATDP and NAT). Comparisons between ATDP and NAT, while clinically relevant, were not the focus of this study, which aimed to elucidate the specific effects of prepregnancy treatment initiation.

IBM SPSS for Windows was utilized for statistical analyses, with statistical significance defined as a two-sided *P* < .05.

### 2.7. Sensitivity analyses

To ensure the robustness of our conclusions, we conducted sensitivity analyses. First, we used propensity scores to match patients receiving and not receiving NA therapy (Table S2, Supplemental Digital Content). Second, we assessed maternal pregnancy outcomes using multivariate binary logistic regression (Table S3, Supplemental Digital Content). Third, we analyzed the risk of abnormal ALT levels by Poisson regression after deleting the data of LdT treatment (Table S4, Supplemental Digital Content). Fourth, we analyzed growth parameters using multivariate linear regression after propensity score matching (Table S5, Supplemental Digital Content). Finally, to assess the impact of missing data handling, we repeated the primary analyses using multiple imputation instead of mean imputation. The results remained consistent, further supporting the robustness of our findings (Tables S10–S12, Supplemental Digital Content). These analyses further validated our findings and strengthened the reliability of our conclusions.

## 3. Results

A retrospective study was conducted encompassing 1205 infants born to mothers with chronic HBV infection, with follow-ups conducted at 1, 3, 6, and 12 months postpartum. A total of 1031 mother-infant pairs were included in the analysis using exclusion criteria and deletion of missing child follow-up data. The cohort was stratified into 3 treatment groups: ATBP (n = 99), ATDP (n = 475), and NAT (n = 457). Maternal ages averaged 31.7 (SD = 3.5) years in the ATBP group, 30.1 (SD = 3.8) years in the ATDP group, and 31.3 (SD = 3.8) years in the NAT group.

Differences in maternal baseline characteristics were observed among groups. Notably, the ATBP cohort demonstrated significantly lower HBV DNA levels and a diminished proportion of individuals with HBV DNA > 5.3 log_10_ IU/mL compared to ATDP and NAT (Table 1). The positive HBeAg rate was 57.6% (57) in the ATBP group, situated between the higher rate in ATDP and the lower rate in NAT. Infant characteristics differed among groups. Prior to matching, significant variations were identified across multiple maternal variables. To address these, 99 patients from each treatment group were matched, resulting in balanced baseline characteristics as presented in Table 2.

**Table 1:** Maternal, delivery, and infant characteristics.

Characteristic	Total	ATBP	ATDP	NAT	*P* *
No. of women	1031	99	475	457	
Age, yr†	30.8 ± 3.8	31.7 ± 3.5	30.1 ± 3.8	31.3 ± 3.8	<.001
<35	876 (85.0)	83 (83.8)	417 (87.8)	376 (82.3)	.059
≥35	155 (15.0)	16 (16.2)	58 (12.2)	81 (17.7)	
BMI, kg/m^2^†,‡	21.8 ± 2.8	21.3 ± 2.9	21.8 ± 2.9	22.0 ± 2.7	.132
<18.5	73 (7.1)	12 (12.1)	34 (7.2)	27 (5.9)	.298
18.5–23.9	775 (75.2)	71 (71.7)	358 (75.4)	346 (75.7)	
24–27.9	153 (14.8)	15 (15.2)	66 (13.9)	72 (15.8)	
≥28	30 (2.9)	1 (1.0)	17 (3.6)	12 (2.6)	
FIB-4†,‡	0.7 ± 0.3	0.7 ± 0.3	0.8 ± 0.4	0.7 ± 0.3	.211
<1.45	1002 (97.2)	97 (98.0)	458 (96.4)	447 (97.8)	.381
1.45–3.25	27 (2.6)	2 (2.0)	17 (3.6)	8 (1.8)	
≥3.25	2 (0.2)	0 (0.0)	0 (0.0)	2 (0.4)	
Gravida§	2 (1, 3)	2 (1, 3)	2 (1, 3)	2 (1, 3)	.036
Primigravida	388 (37.6)	28 (28.3)	198 (41.7)	162 (35.5)	.019
Parity§	0 (0, 1)	1 (0, 1)	0 (0, 1)	0 (0, 1)	.020
Primiparity	585 (56.7)	45 (45.5)	291 (61.3)	249 (54.5)	.007
Cesarean history	211 (20.5)	22 (22.2)	88 (18.5)	101 (22.1)	.361
Positive HBeAg	489 (47.4)	57 (57.6)	385 (81.1)	47 (10.3)	<.001
HBV DNA, log_10_ IU/mL†	5.1 ± 2.3	2.9 ± 0.8	7.2 ± 1.4	3.4 ± 1.2	<.001
>5.3 log_10_ IU/mL, no. (%)	429 (41.61)	2 (2.0)	402 (84.6)	25 (5.5)	<.001
Antiviral drug					<.001
LdT	52 (5.0)	5 (5.1)	47 (9.9)	NA	
TDF	491 (47.6)	85 (85.9)	406 (85.5)	NA	
TAF	31 (3.0)	9 (9.1)	22 (4.6)	NA	
Delivery					
Type of delivery, no. (%)					.584
Vaginal	528 (51.2)	51 (51.5)	251 (52.8)	226 (49.5)	
Cesarean section	503 (48.8)	48 (48.5)	224 (47.2)	231 (50.6)	
Gestational age, wk†	39.1 ± 1.2	39.1 ± 1.4	39.1 ± 1.3	39.1 ± 1.1	.700
Distribution, no. (%)					.260
<32 wk	3 (0.3)	1 (1.0)	2 (0.4)	0 (0.0)	
≥32 to <35 wk	7 (0.7)	0 (0.0)	3 (0.6)	4 (0.9)	
≥35 to <37 wk	30 (2.9)	4 (4.0)	17 (3.6)	9 (2.0)	
≥37 wk	991 (96.1)	94 (95.0)	453 (95.4)	444 (97.2)	
Infants					
Sex, no. (%)					.728
Male	515 (50.0)	50 (50.5)	243 (51.2)	222 (48.6)	
Female	516 (50.0)	49 (49.5)	232 (48.8)	235 (51.4)	
Apgar score at 1 min, no. (%)					.045
0–3	1 (0.1)	1 (1.0)	0 (0.0)	0 (0.0)	
4–6	2 (0.2)	0 (0.0)	0 (0.0)	2 (0.4)	
7–10	1028 (99.7)	98 (99.0)	475 (100.0)	455 (99.6)	
Birth weight, kg†	3.3 ± 0.4	3.3 ± 0.4	3.3 ± 0.4	3.4 ± 0.4	<.001
Birth height, cm†	50.2 ± 1.2	50.1 ± 1.3	50.1 ± 1.2	50.3 ± 1.1	.280

ANOVA = analysis of variance, ATBP = antiviral treatment before pregnancy, ATDP = antiviral treatment during pregnancy, BMI = body mass index, HBeAg = hepatitis B e antigen, IQR = interquartile range, LdT = telbivudine, NAT = no antiviral treatment, SD = standard deviation, TAF = tenofovir alafenamide fumarate, TDF = tenofovir disoproxil fumarate.

* Quantitative data that were normally distributed were analyzed via ANOVA and non-normally distributed data were analyzed with the Kruskal–Wallis test; categorical data were examined with the chi-square test.

† Mean ± SD.

‡ The percentage of missing values was 1.9% for FIB-4 and 17.18% for BMI.

§ Median (IQR).

**Table 2:** Baseline characteristics after propensity score matching.

Characteristic	ATBP	ATDP	*P* *	NAT	*P* †
No. of women with data	99	99		99	
Age, yr‡	31.7 ± 3.5	29.9 ± 3.7	<.001	31.1 ± 4.0	.253
<35	83 (83.8)	89 (89.9)	.207	80 (80.8)	.576
≥35	16 (16.2)	10 (10.1)		19 (19.2)	
BMI, kg/m^2^‡	21.3 ± 2.9	21.1 ± 2.8	.570	21.4 ± 2.6	.937
<18.5	12 (12.1)	16 (16.2)	.588	11 (11.1)	.946
18.5–23.9	71 (71.7)	68 (68.7)		74 (74.8)	
24–27.9	15 (15.2)	12 (12.1)		13 (13.1)	
≥28	1 (1.0)	3 (3.0)		1 (1.0)	
FIB-4	0.7 ± 0.3	0.8 ± 0.4	.063	0.8 ± 0.4	.431
<1.45	97 (98.0)	95 (96.0)	.678	97 (97.98)	1.000
1.45–3.25	2 (2.0)	4 (4.0)		1 (1.0)	
≥3.25	0 (0.0)	0 (0.0)		1 (1.0)	
Gravida§	2 (1, 3)	2 (1, 3)	.578	2 (1, 3)	.807
Gravida = 1	28 (28.3)	30 (30.3)	.755	29 (29.3)	.875
Parity§	1 (0, 1)	0 (0, 1)	1.000	1 (0, 1)	.997
Parity = 0	45 (45.5)	45 (45.5)	1.000	45 (45.5)	1.000
Cesarean history	22 (22.2)	27 (27.3)	.410	29 (29.3)	.255
Positive HBeAg	57 (57.6)	87 (87.9)	<.001	9 (9.1)	<.001
HBV DNA, log_10_ IU/mL‡	2.9 ± 0.8	7.2 ± 1.4	<.001	3.6 ± 1.4	<.001
>5.3 log_10_ IU/mL, no. (%)	2 (2.0)	86 (86.9)	<.001	11 (11.1)	.010
GA < 24 wk‡	19.1 ± 4.3	19.9 ± 4.6	.185	20.3 ± 4.1	.043
ALT levels (<24 wk), U/L§	15.6 (11.9, 20.0)	23.9 (17.9, 39.0)	<.001	17.2 (11.5, 22.4)	.330
Antiviral drug					
LdT	5 (5.1)	35 (35.4)	<.001	NA	NA
TDF	85 (85.9)	63 (63.6)		NA	
TAF	9 (9.1)	1 (1.0)		NA	
GA > 28 wk‡	34.4 ± 2.4	33.8 ± 2.2	.114	34.3 ± 2.3	.914
ALT levels (>28 wk), U/L§	13.4 (10.0, 15.8)	16.0 (13.6, 22.2)	<.001	12.3 (9.4, 15.8)	.341
Delivery					
Type of delivery, no. (%)					
Vaginal	51 (51.5)	48 (48.5)	.670	47 (47.5)	.570
Cesarean section	48 (48.5)	51 (51.5)		52 (52.5)	
Gestational age, wk‡	39.1 ± 1.4	39.0 ± 1.1	.421	39.0 ± 1.2	.401
Distribution, no. (%)					
<32 wk	1 (1.0)	0 (0.0)	1.000	0 (0.0)	.446
≥32 to <35 wk	0 (0.0)	0 (0.0)		2 (2.0)	
≥35 to <37 wk	4 (4.0)	4 (4.0)		2 (2.0)	
≥37 wk	94 (95.0)	95 (96.0)		95 (96.0)	
Infants					
Sex, no. (%)					
Male	50 (50.5)	43 (43.4)	.319	54 (54.5)	.569
Female	49 (49.5)	56 (56.6)		45 (45.5)	
Apgar score at 1 min, no. (%)					
0–3	1 (1.0)	0 (0.0)	1.000	0 (0.0)	1.000
4–6	0 (0.0)	0 (0.0)		0 (0.0)	
7–10	98 (99)	99 (100.0)		99 (100.0)	
Birth weight, kg‡	3.3 ± 0.4	3.3 ± 0.4	.631	3.4 ± 0.4	.142
Birth height, cm‡	50.1 ± 1.3	50.2 ± 0.9	.228	50.3 ± 1.1	.084

ATBP = antiviral treatment before pregnancy, ATDP = antiviral treatment during pregnancy, BMI = body mass index, HBeAg = hepatitis B e antigen, IQR = interquartile range, LdT = telbivudine, NAT = no antiviral treatment, SD = standard deviation, TAF = tenofovir alafenamide fumarate, TDF = tenofovir disoproxil fumarate.

* ATBP versus ATDP.

† ATBP versus NAT.

‡ Mean ± SD.

§ Median (IQR).

### 3.1. Maternal pregnancy outcomes

Prior to assessing the value of prepregnancy treatment, we evaluated the established standard of care. In the matched cohort, ATDP was associated with significantly lower ALT levels before 24 weeks of gestation compared to NAT (adjusted mean difference = −16.63 U/L, 95% CI = −26.38 to −6.89; *P* = .001; Table S8, Supplemental Digital Content). Regarding the clinical outcome, the risk of gestational ALT abnormality was lower in the ATDP group, though not statistically significant after adjustment (adjusted relative risk [aRR] = 0.51, 95% CI = 0.25–1.05; *P* = .067; Table S9, Supplemental Digital Content). Against this background, we focused on evaluating the incremental value of ATBP. Table 3 displays the correlation of the ALT levels at various gestational ages between matched groups. In all 3 groups, ALT levels tended to decrease from the second trimester to the third trimester. The median ALT level in the ATBP group decreased from 15.6 U/L (IQR = 11.9–20.0) at <24 weeks to 13.4 U/L (IQR = 10.0–15.8) at >28 weeks; similar trends were observed in the ATDP and NAT groups (Table 2). In the adjusted model, the ATBP group had significantly lower ALT levels before 24 gestational weeks when compared to the ATDP (estimate [E] = −18.81, *P* < .001) and NAT groups (E = −4.62, *P* = .048). However, in the adjusted model, no statistically significant difference was observed in ALT levels after 28 gestational weeks when comparing the ATBP group to the ATDP group (E = −18.66, *P* = .083) or to the NAT group (E = −1.01, *P* = .761).

**Table 3:** ALT levels analyzed by multivariate linear regression.*

Comparison	Gestational period	Model	E (95% CI) U/L	*P* value
ATBP vs ATDP	GA < 24 wk	Crude	−20.21 (−28.98 to −11.44)	<.001
		Adjusted*	−18.81 (−27.10 to −10.52)	<.001
	GA > 28 wk	Crude	−17.66 (−37.15 to 1.83)	.076
		Adjusted*	−18.66 (−39.75 to 2.42)	.083
ATBP vs NAT	GA < 24 wk	Crude	−4.46 (−9.12 to 0.20)	.061
		Adjusted*	−4.62 (−9.20 to −0.03)	.048
	GA > 28 wk	Crude	−1.00 (−7.45 to 5.45)	.761
		Adjusted*	−1.01 (−7.55 to 5.53)	.761

ALT = alanine aminotransferase, ATBP = antiviral treatment before pregnancy, ATDP = antiviral treatment during pregnancy, BMI = body mass index, CI = confidence interval, E = estimate, GA = gestational age, NAT = no antiviral treatment.

* Multivariate linear regression was adjusted for maternal age, BMI, primigravida, and primiparity.

**Figure 1.**
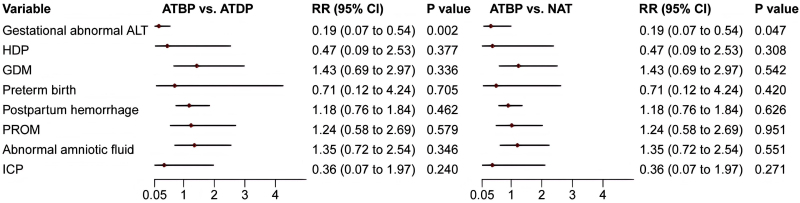
Pregnancy outcomes after propensity score matching. ALT = alanine aminotransferase, ATBP = antiviral treatment before pregnancy, ATDP = antiviral treatment during pregnancy, CI = confidence interval, GDM = gestational diabetes mellitus, HDP = hypertensive disorders of pregnancy, ICP = intrahepatic cholestasis of pregnancy, NAT = no antiviral treatment, PROM = premature rupture of the membranes, RR = relative risk.

Figure 1 and Table S6, Supplemental Digital Content present the comparative maternal pregnancy outcomes across matched groups. A marked and statistically significant decline in gestational ALT abnormalities was observed in the ATBP group. Compared to the ATDP group, the aRR was 0.19 (95% CI = 0.07–0.55; *P* = .002); compared to the NAT group, the aRR was 0.34 (95% CI = 0.12–0.99; *P* = .047). The prevalence was 4.0% in the ATBP group, versus 22.2% and 12.1% in the ATDP and NAT groups, respectively. Conversely, no statistically significant differences emerged in the rates of HDP, GDM, preterm birth, postpartum hemorrhage, PROM, abnormal amniotic fluid indices, or ICP between the ATBP group and its counterparts in the ATDP and NAT groups.

### 3.2. Growth parameters of offspring

Infants of varying sexes were tracked from birth through 1 year of age to assess their physical growth and development trajectories. No significant differences were observed in Apgar scores, birth weight, or height in the ATBP group compared to the ATDP and NAT groups. Utilizing age- and sex-specific *Z* scores, there were no significant disparities in WAZ, HAZ, or the rates of tooth eruption and fontanel closure (Table S5, Supplemental Digital Content).

**Figure 2.**
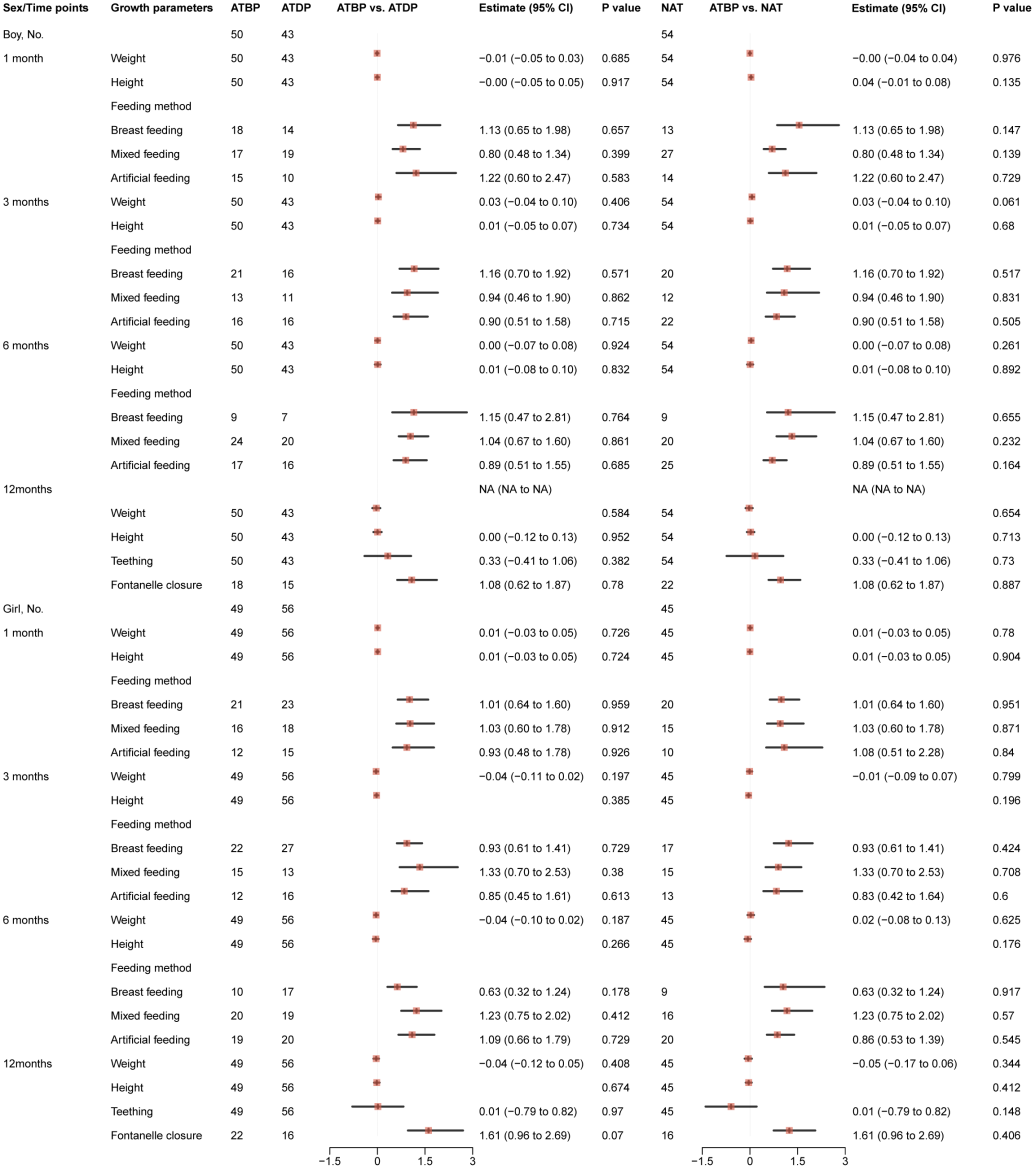
Growth parameters of children stratified by subgroup. ATBP = antiviral treatment before pregnancy, ATDP = antiviral treatment during pregnancy, CI = confidence interval, NAT = no antiviral treatment.

The growth of infants in different groups stratified by sex is shown in Figure 2 and Table S7, Supplemental Digital Content. Boys were more likely to be breastfed at 1, 3, and 6 months of age in the ATBP group. Boys had more teeth, and girls had fewer in the ATBP group at 12 months compared to the ATDP group. Both boys and girls had a higher proportion of fontanel closure, although there was no statistically significant difference in these indicators. In addition, the difference in weight and height was not observed between the 2 groups.

All infants received the recommended schedule of HBV vaccination and hepatitis B immunoglobulin. In this study, only 1 infant from the ATDP group (receiving antiviral treatment from 24 weeks of gestation) was identified as having HBV infection at 7 to 12 months of age.

## 4. Discussion

Our study aimed to elucidate the influence of ATBP on follow-up outcomes among individuals with chronic HBV infection. Compared to those in the ATDP and NAT groups, participants administered ATBP exhibited a decreased risk of gestational abnormal ALT levels. However, no significant differences were observed in WAZ, HAZ, or rates of tooth eruption and fontanel closure.

The optimal timing of antiviral therapy poses a challenge for pregnant women with chronic HBV infection, stemming from uncertainties surrounding this clinical scenario. Current clinical trials primarily focus on mitigating mother-to-child transmission^[21,22]^ and assessing drug efficacy and safety.^[23]^ International guidelines recommend therapeutic intervention in individuals who have a family history of liver cirrhosis or hepatocellular carcinoma, are aged over 30, exhibit significant hepatic inflammation or fibrosis, and manifest HBV-related extrahepatic manifestations.^[13,24]^ Despite the growing trend of women receiving NA therapy before pregnancy, research on follow-up outcomes remains scarce for this population.

The data derive from multiple studies on pregnancy management. A prospective study underscored the safety and efficacy of TAF or TDF throughout or beginning in early pregnancy for women with active hepatitis and their offspring.^[25]^ This investigation specifically targeted pregnant women experiencing active hepatitis before the first trimester of pregnancy. Another retrospective analysis encompassing 165 pregnant women revealed that TDF or LdT therapy did not adversely impact the obstetrical outcomes of mothers or the long-term physical and skeletal development of children under 5 years,^[26]^ albeit with a modest sample size. Our findings echoed these previous studies, demonstrating no elevated risk of adverse pregnancy outcomes. However, although ATBP significantly reduced the risk of gestational ALT abnormalities, this benefit did not translate into statistically significant differences in other maternal clinical outcomes such as HDP, GDM, preterm birth, or postpartum hemorrhage. This may be due to insufficient statistical power for these secondary outcomes or because ALT flares in this low-risk cohort were mostly mild and did not progress to overt clinical events. Furthermore, we observed a decreased risk of gestational abnormal ALT levels in women with NA therapy prior to conception, which may be attributed to the low viral burden of mothers at the early stages of pregnancy. This is also crucial in reducing the risk of mother-to-child transmission. The potential benefits of antiviral intervention include suppressing virus replication, reducing inflammatory processes in the liver, reversing the development of fibrosis, and preventing the development of cirrhosis and hepatocellular carcinoma. Thus, to identify changes in the disease state early on, women with chronic HBV infection should undergo more frequent serological testing during pregnancy, such as liver function and quantitative HBV DNA tests.

Pregnant women with high HBV DNA levels exhibited a heightened risk of gestational ALT flares.^[27,28]^ ALT elevations have been documented in both nonpregnant and pregnant HBV patients following antiviral medications.^[22,29,30]^ Moreover, cessation of antiviral therapy during pregnancy has been identified as a risk for ALT flares in mothers.^[31]^ This increased risk of gestational abnormal ALT is associated with preconception high viral loads, ATDP, or treatment discontinuation. For women undergoing long-term NA therapy contemplating or confirming pregnancy, maintaining treatment may mitigate the risk of gestational ALT flares. However, further research is warranted to elucidate the underlying mechanisms mediating this potential benefit. According to the disease condition, patients on ATDP for the purpose of preventing mother-to-child transmission could be discontinued after childbirth. Maternal ALT levels should be monitored every 2 to 3 months for the first 6 months after withdrawal.

Infants exposed to TDF frequently display transient growth retardation due to renal and skeletal toxicities, yet clinical evidence remains contentious. Gender-specific variations in infant growth parameters, including weight and length, are well recognized.^[32]^ In our investigation, TDF utilization surpassed 80% among NA therapy recipients. Importantly, reassuring evidence is emerging from long-term studies within the HBV field itself. For instance, a prospective cohort study with over 6 years of follow-up found no adverse effects of maternal TDF exposure on children’s growth, bone, or renal outcomes; this is corroborated by a large-scale study confirming TDF’s long-term infant safety, and further supported by a 10-year follow-up study demonstrating the safety profile of LdT used during pregnancy.^[23,33,34]^ Building upon this primary evidence base, insights from HIV cohort studies offer additional perspectives on potential mechanisms. Insights into the impact of TDF on infants can be garnered from HIV cohort studies. Breast milk calcium concentrations peaked at 14 weeks postpartum in HIV-positive mothers on TDF-based antiretrovirals, subsequently declining.^[35,36]^ Low maternal serum calcium levels were associated with reduced weight and length,^[37]^ suggesting a potential link between medication-modulated milk calcium and infant growth. Remarkably, in children exposed to HIV and antiretrovirals in utero, growth and development were comparable, with breastfeeding universally enhancing weight, height, and overall development, regardless of maternal HIV status.^[38]^ In an Asian population study, maternal TDF use did not correlate with growth retardation or abnormal bone findings, nor was it associated with infant transient rickets, growth impairment, or renal dysfunction.^[39]^ Despite these insights, our results failed to detect gender-specific disparities in growth parameters among infants in the ATBP group compared to those in the ATDP and NAT groups. These findings warrant further investigation to confirm their validity and explore potential underlying mechanisms.

The observation that significantly more women initiated LdT during pregnancy than before is indeed a noteworthy finding. More importantly, this observed difference in drug regimen does not confound our primary finding regarding the benefit of preconception therapy. The reduction in gestational ALT abnormalities associated with ATBP is unlikely to be solely attributable to the specific antiviral agents used, as it was evident even in early pregnancy (<24 weeks of gestation; Table 3) and remained statistically significant in a rigorous sensitivity analysis that excluded all patients who received LdT (Table S4, Supplemental Digital Content). This trend might be attributed to several factors. On the one hand, switching to TDF treatment before pregnancy is recommended for patients with long-term LdT if resistance develops.^[40,41]^ On the other hand, doctors and patients might choose a more cautious approach because of concerns about the potential drug safety or the lack of solid data to support the use of LdT beginning in early pregnancy. Furthermore, TDF use during pregnancy has shown better long-term efficacy than LdT, while neither has been shown to impair mothers or the long-term development of the child. In our study, fewer women started LdT before pregnancy, which might introduce bias in our findings. To address this, we removed LdT treatment data from the analysis, and the results remained reliable. This finding underscores the need, in clinical practice, for physicians to consider individualized risks and benefits when choosing treatment options for patients and to seek more cautious alternatives when necessary.

Building on these findings, our future research will address the need for long-term (>5 years) data through a dedicated prospective study. This will involve extending follow-up of children to school age (5–7 years), expanding recruitment via a multicenter design to include a larger sample (target > 300), and evaluating comprehensive outcomes, including neurodevelopment and bone health.

The present study encounters several limitations. Firstly, the absence of variables in the databases poses a challenge in assessing nutritional status, such as calcium and vitamin D levels, along with records of supplement usage. Secondly, the study’s reliance on less detailed data, such as the timing of feeding method changes and potential growth parameter measurement errors, necessitates more detailed information collection. Thirdly, the retrospective nature of the study restricts the number of mothers receiving NAs therapy before pregnancy, screening the broader population of HBV-infected individuals, both retrospectively and prospectively, to enhance sample size and facilitate longer-term follow-ups. Fourthly, the comparison of optimal therapeutic agents, which are often influenced by individual preferences, requires clarification to ensure treatment efficacy is accurately evaluated. Fifthly, the sample size of this study was determined based on the primary outcome (gestational abnormal ALT). Consequently, for some secondary outcomes, particularly the analysis of infant growth parameters after stratification by sex, the statistical power is relatively limited and is more suited to ruling out large between-group differences. The nonsignificant trends observed in our report warrant further validation in prospective studies or larger cohorts. Sixthly, an infant loss-to-follow-up rate of 14.4% introduces a risk of attrition bias. Our conclusions about infant growth are based on infants who completed follow-up. If those lost to follow-up had systematically different growth outcomes, and if this difference was not balanced across the maternal treatment groups, then our between-group comparisons could be biased. Although baseline maternal characteristics were comparable (Table S1, Supplemental Digital Content), we cannot exclude this possibility. Seventhly, this study has limitations inherent to its observational and single-center design. Despite propensity score matching, residual confounding by unmeasured factors (e.g., detailed nutritional status or medication adherence) cannot be excluded. Furthermore, as the study was conducted in a region with high HBV endemicity (prevalence ~5.6% in China), the generalizability of findings to populations with substantially different epidemiological patterns (e.g., the global prevalence of ~3.8%) or healthcare settings may be limited. We also lack preconception ALT data, which prevents a full assessment of ALT changes from before pregnancy. Future multicenter studies involving diverse populations are warranted to validate our findings. Lastly, while rigorous statistical methods aim to mitigate bias and confounding, cohort studies inherently carry the risk of bias and confounding. To address these limitations, future endeavors should strive to include patients with only chronic HBV infection, enhancing the generalizability of findings while adopting strategies to mitigate biases and augment data granularity.

## 5. Conclusions

HBsAg should be screened in women of childbearing age who are preparing for pregnancy. HBV DNA and liver function should be tested in HBsAg-positive women. In our investigation, preconception administration of NA therapy was associated with a lower risk of maternal ALT abnormalities, whereas we did not find a difference in maternal pregnancy outcomes or infant growth parameters in the first year of life. This highlights the importance of specialized counseling and prenatal planning to maximize health for women with chronic HBV infection.

## Author contributions

**Conceptualization:** Xing-Ran Tao, Guorong Han.

**Formal analysis:** Xing-Ran Tao.

**Visualization:** Xing-Ran Tao, Jia Jia.

**Writing – original draft:** Xing-Ran Tao.

**Writing – review & editing:** Xing-Ran Tao, Shi-Jing Gao, Jia Jia.

**Investigation:** Shi-Jing Gao.

**Validation:** Shi-Jing Gao.

**Data curation:** Jia Jia.

**Funding acquisition:** Guorong Han.

**Resources:** Guorong Han.

**Supervision:** Guorong Han.

























**Figure:**