# Supplementary material for: Maternal and infant growth outcomes following preconception antiviral therapy in chronic hepatitis B virus infection: A retrospective cohort study
Source: Medicine (Baltimore). 2026 Jun 12;105(24):e49131. doi: 10.1097/MD.0000000000049131 (PMC13268500; doi:10.1097/MD.0000000000049131)
Supplement: Supplementary file 1 [file medi-105-e49131-s002.docx]

| Supplementary Table 1. Sensitivity analysis between patients with vs. without the follow-up data of their infants | | | |
| --- | --- | --- | --- |
| Characteristic, no. (%) | Patients with missing follow-up data N=174 | Patients with known follow-up data N=1031 | P |
| Age, y ^a^ | 29.7 ± 4.1 | 30.8 ± 3.8 | 0.001 |
| <35 | 155 (89.1) | 876 (85.0) | 0.163 |
| ≥35 | 19 (10.9) | 155 (15.0) |  |
| BMI, kg/m^2^ ^a^ | 21.7 ± 2.4 | 21.8 ± 2.8 | 0.442 |
| <18.5 | 13 (7.5) | 73 (7.1) | 0.001 |
| 18.5-23.9 | 147 (84.5) | 775 (75.2) |  |
| 24-27.9 | 6 (3.4) | 153 (14.8) |  |
| ≥28 | 8 (4.6) | 30 (2.9) |  |
| Gravida ^b^ | 2 (1,3) | 2 (1,3) | 0.294 |
| Gravida=1 | 62 (35.6) | 388 (37.6) |  |
| Parity ^b^ | 0 (0, 1) | 0 (0, 1) | 0.096 |
| Parity=0 | 91 (52.3) | 585 (56.7) | 0.284 |
| Caesarean history | 43 (24.7) | 211 (20.5) | 0.228 |
| Positive HBeAg | 107 (61.5) | 489 (47.4) | <0.001 |
| HBV DNA, log_10_ IU/ml ^a^ | 5.4 ± 2.4 | 5.1 ± 2.3 | 0.720 |
| >5.3 log_10_ IU/ml, no. (%) | 86 (49.4) | 429 (41.6) | 0.055 |
| Antiviral treatment |  |  |  |
| Before pregnancy | 17 (9.8) | 99 (9.6) | 0.158 |
| During pregnancy | 93 (53.4) | 475 (46.1) |  |
| None | 64 (36.8) | 457 (44.3) |  |
| Antiviral drug |  |  |  |
| LdT | 16 (9.2) | 52 (5.0) | 0.074 |
| TDF | 90 (51.7) | 492 (47.7) |  |
| TAF | 4 (2.3) | 31 (3.0) |  |

BMI, body mass index; HBeAg, hepatitis B e antigen; LdT, telbivudine; TDF, tenofovir disoproxil fumarate; TAF, tenofovir alafenamide fumarate; SD, standard deviation; IQR, interquartile range.

^a^ Mean ± SD.

^b^ Median (IQR).
