# Supplementary material for: Maternal and infant growth outcomes following preconception antiviral therapy in chronic hepatitis B virus infection: A retrospective cohort study
Source: Medicine (Baltimore). 2026 Jun 12;105(24):e49131. doi: 10.1097/MD.0000000000049131 (PMC13268500; doi:10.1097/MD.0000000000049131)
Supplement: Supplementary file 2 [file medi-105-e49131-s003.docx]

| Supplementary Table 2. Outcomes compared between with or without NA therapy after propensity score matching | | | | |
| --- | --- | --- | --- | --- |
|  | NA therapy ^a^ | Without NA therapy | RR (95%CI) | P ^b^ |
| Mothers | 448 | 448 |  |  |
| Abnormal ALT | 83 (18.5) | 42 (9.4) | 1.99 (1.41, 2.81) | <0.001 |
| HDP | 15 (3.3) | 11 (2.5) | 1.37 (0.64, 2.94) | 0.415 |
| GDM | 69 (15.4) | 79 (17.6) | 0.88 (0.66, 1.12) | 0.388 |
| Preterm birth | 12 (2.7) | 7 (1.6) | 1.78 (0.71, 4.46) | 0.219 |
| Postpartum hemorrhage | 113 (25.2) | 146 (32.6) | 0.78 (0.63, 0.96) | 0.017 |
| PROM | 82 (18.3) | 66 (14.7) | 1.26 (0.94, 1.69) | 0.124 |
| Abnormal amniotic fluid | 70 (15.6) | 59 (13.2) | 1.19 (0.86, 1.63) | 0.297 |
| ICP | 14 (3.1) | 14 (3.1) | 1.00 (0.49, 2.05) | 0.996 |

NA, nucleos(t)ide analogue; RR, relative risk; CI, confidence interval; ALT, alanine aminotransferase; HDP, hypertensive disorders of pregnancy; GDM, gestational diabetes mellitus; PROM, premature rupture of the membranes; ICP, intrahepatic cholestasis of pregnancy; BMI, body mass index.

^a^ NA therapy included ATBP and ATDP groups.^b^ Multivariate analyses were adjusted for maternal age, BMI, primigravida, primiparity by Poisson regression.
