# Supplementary material for: Maternal and infant growth outcomes following preconception antiviral therapy in chronic hepatitis B virus infection: A retrospective cohort study
Source: Medicine (Baltimore). 2026 Jun 12;105(24):e49131. doi: 10.1097/MD.0000000000049131 (PMC13268500; doi:10.1097/MD.0000000000049131)
Supplement: Supplementary file 3 [file medi-105-e49131-s004.docx]

| Supplementary Table 3. Outcomes analyzed by binary logistic regression ^a^ | | | | | | | |
| --- | --- | --- | --- | --- | --- | --- | --- |
| Variables | ATBP  N=99 | ATDP  N=475 | Adjusted model | | NAT  N=457 | Adjusted model | |
|  |  |  | OR (95%CI) | P ^b^ |  | OR (95%CI) | P ^c^ |
| Gestational abnormal ALT | 4 (4.0) | 106 (22.3) | 0.15 (0.05, 0.41) | <0.001 | 43 (9.4) | 0.41 (0.14, 1.16) | 0.091 |
| HDP | 2 (2.0) | 16 (3.4) | 0.58 (0.13, 2.63) | 0.479 | 11 (2.4) | 0.84 (0.18, 3.83) | 0.818 |
| GDM | 15 (15.2) | 69 (14.5) | 1.01 (0.54, 1.88) | 0.976 | 81 (17.7) | 0.83 (0.46, 1.51) | 0.540 |
| Preterm birth | 2 (2.0) | 16 (3.4) | 0.70 (0.15, 3.24) | 0.648 | 7 (1.5) | 1.33 (0.27, 6.48) | 0.657 |
| Postpartum hemorrhage | 30 (30.3) | 109 (22.9) | 1.51 (0.92, 2.47) | 0.105 | 149 (32.6) | 0.90 (0.56, 1.44) | 0.628 |
| PROM | 14 (14.1) | 86 (18.1) | 0.83 (0.44, 1.57) | 0.570 | 67 (14.7) | 0.96 (0.51, 1.79) | 0.894 |
| Abnormal amniotic fluid | 18 (18.2) | 71 (14.9) | 1.34 (0.74, 2.42) | 0.336 | 60 (13.1) | 1.47 (0.82, 2.62) | 0.191 |
| ICP | 2 (2.0) | 14 (3.0) | 0.83 (0.18,3.85) | 0.810 | 14 (3.1) | 0.65 (0.15, 2.92) | 0.576 |

ATBP, antiviral treatment before pregnancy; ATDP, antiviral treatment during pregnancy; NAT, no antiviral treatment; OR, odds ratio; CI, confidence interval; ALT, alanine aminotransferase; HDP, hypertensive disorders of pregnancy; GDM, gestational diabetes mellitus; PROM, premature rupture of the membranes; ICP, intrahepatic cholestasis of pregnancy; BMI, body mass index.

a Multivariate analyses were adjusted for maternal age, BMI, primigravida, primiparity by binary logistic regression.

b ATBP vs. ATDP

c ATBP vs. NAT
