# Supplementary material for: Maternal and infant growth outcomes following preconception antiviral therapy in chronic hepatitis B virus infection: A retrospective cohort study
Source: Medicine (Baltimore). 2026 Jun 12;105(24):e49131. doi: 10.1097/MD.0000000000049131 (PMC13268500; doi:10.1097/MD.0000000000049131)
Supplement: Supplementary file 4 [file medi-105-e49131-s005.docx]

Supplementary Table 4. Abnormal ALT levels analyzed by Poisson regression after deleting the data of LdT treatment ^a^

| Variable | n (%) | Crude model | | Adjusted model | |
| --- | --- | --- | --- | --- | --- |
|  |  | RR (95%CI) | P | RR (95%CI) | P |
| ATBP | 3 (3.2) | Reference |  | Reference |  |
| ATDP | 97 (22.7) | 7.10 (2.30, 21.92) | 0.001 | 7.37 (2.33, 23.29) | 0.001 |
| NAP | 42 (9.2) | 2.88 (0.91, 9.10) | 0.071 | 2.72 (0.86, 8.60) | 0.089 |

LdT, telbivudine; ALT, alanine aminotransferase; ATBP, antiviral treatment before pregnancy; ATDP, antiviral treatment during pregnancy; NAT, no antiviral treatment; RR, relative risk; CI, confidence interval; BMI, body mass index.

^a^ Multivariate analyses were adjusted for maternal age, BMI, primigravida, primiparity by Poisson regression.
