# Supplementary material for: Maternal and infant growth outcomes following preconception antiviral therapy in chronic hepatitis B virus infection: A retrospective cohort study
Source: Medicine (Baltimore). 2026 Jun 12;105(24):e49131. doi: 10.1097/MD.0000000000049131 (PMC13268500; doi:10.1097/MD.0000000000049131)
Supplement: Supplementary file 5 [file medi-105-e49131-s006.docx]

| Supplementary Table 5. Growth parameters of children analyzed by linear regression with multiple variables ^a^ | | | | | |
| --- | --- | --- | --- | --- | --- |
| Variables | ATBP | ATDP | P ^b^ | NAT | P ^c^ |
| Children | 99 | 99 |  | 99 |  |
| 1 month |  |  |  |  |  |
| Weight | 1.89 ± 0.23 | 1.91 ± 0.13 | 0.869 | 1.92 ± 0.12 | 0.891 |
| Height | 1.95 ± 0.24 | 1.97 ± 0.13 | 0.760 | 1.96 ± 0.14 | 0.368 |
| 3 months |  |  |  |  |  |
| Weight | 1.48 ± 0.26 | 1.50 ± 0.18 | 0.822 | 1.48 ± 0.18 | 0.407 |
| Height | 1.74 ± 0.25 | 1.77 ± 0.15 | 0.798 | 1.78 ± 0.16 | 0.468 |
| 6 months |  |  |  |  |  |
| Weight | 1.62 ± 0.18 | 1.64 ± 0.18 | 0.413 | 1.59 ± 0.23 | 0.308 |
| Height | 1.28 ± 0.30 | 1.33 ± 0.23 | 0.454 | 1.33 ± 0.23 | 0.338 |
| 12months |  |  |  |  |  |
| Weight | 1.16 ± 0.32 | 1.20 ± 0.24 | 0.267 | 1.20 ± 0.23 | 0.425 |
| Height | 1.30 ± 0.30 | 1.32 ± 0.27 | 0.729 | 1.32 ± 0.23 | 0.924 |
| Teething | 6.83 ± 2.19 | 6.69 ± 1.80 | 0.563 | 7.11 ± 2.23 | 0.735 |
| Fontanelle closure, no. | 40 (44.4) | 31 (31.3) | 0.125 | 38 (38.4) | 0.498 |

ATBP, antiviral treatment before pregnancy; ATDP, antiviral treatment during pregnancy; NAT, no antiviral treatment.

a Multivariate analyses were adjusted for weight (birth), height (birth) and feeding method at 1, 3 and 6 months and adjusted for weight (birth), height (birth) at 12 months. Weight and height were transformed to Z-scores of weight-for-age and height-for-age, using Ln (Z score-min+1) for analysis.

b ATBP vs. ATDP

c ATBP vs. NAT
