# Supplementary material for: Maternal and infant growth outcomes following preconception antiviral therapy in chronic hepatitis B virus infection: A retrospective cohort study
Source: Medicine (Baltimore). 2026 Jun 12;105(24):e49131. doi: 10.1097/MD.0000000000049131 (PMC13268500; doi:10.1097/MD.0000000000049131)
Supplement: Supplementary file 6 [file medi-105-e49131-s007.docx]

| Supplementary Table 10. ALT levels analyzed by multivariate linear regression ^a^ | | | | |
| --- | --- | --- | --- | --- |
| Comparison | Gestational Period | Model | E (95% CI)U/L | P value |
| ATBP vs. ATDP | GA < 24 weeks | Crude | -20.211 (-29.079, -11.343) | <0.001 |
|  |  | Adjusted* | -20.308 (-29.502, -11.113) | <0.001 |
|  | GA > 28 weeks | Crude | -16.279 (-35.970, 3.411) | 0.105 |
|  |  | Adjusted* | -20.165 (-40.391, 0.060) | 0.051 |
| ATBP vs. NAT | GA < 24 weeks | Crude | -4.459 (-9.172, 0.255) | 0.064 |
|  |  | Adjusted* | -4.927 (-9.663, -0.192) | 0.041 |
|  | GA > 28 weeks | Crude | -1.002 (-7.525, 5.521) | 0.762 |
|  |  | Adjusted* | -1.091 (-7.709, 5.526) | 0.745 |

ATBP, antiviral treatment before pregnancy; ATDP, antiviral treatment during pregnancy; NAT, no antiviral treatment; E, estimate; CI, confidence interval; ALT, alanine aminotransferase; GA, gestational age; BMI, body mass index.

a Multivariate linear regression was adjusted for maternal age, BMI, primigravida, primiparity.
