# Supplementary material for: Maternal and infant growth outcomes following preconception antiviral therapy in chronic hepatitis B virus infection: A retrospective cohort study
Source: Medicine (Baltimore). 2026 Jun 12;105(24):e49131. doi: 10.1097/MD.0000000000049131 (PMC13268500; doi:10.1097/MD.0000000000049131)
Supplement: Supplementary file 7 [file medi-105-e49131-s008.docx]

| Supplementary Table 8. ALT levels analyzed by multivariate linear regression ^a^ | | | | |
| --- | --- | --- | --- | --- |
| Comparison | Gestational Period | Model | E (95% CI)U/L | P value |
| ATDP vs. NAT | GA < 24 weeks | Crude | -15.75 (-25.35, -6.16) | 0.001 |
|  |  | Adjusted* | -16.63 (-26.38, -6.89) | 0.001 |
|  | GA > 28 weeks | Crude | -16.66 (-36.98, 3.66) | 0.107 |
|  |  | Adjusted* | -18.99 (-39.51, 1.53) | 0.069 |

ATBP, antiviral treatment before pregnancy; ATDP, antiviral treatment during pregnancy; NAT, no antiviral treatment; E, estimate; CI, confidence interval; ALT, alanine aminotransferase; GA, gestational age; BMI, body mass index.

a Multivariate linear regression was adjusted for maternal age, BMI, primigravida, primiparity.
