# Supplementary material for: Maternal and infant growth outcomes following preconception antiviral therapy in chronic hepatitis B virus infection: A retrospective cohort study
Source: Medicine (Baltimore). 2026 Jun 12;105(24):e49131. doi: 10.1097/MD.0000000000049131 (PMC13268500; doi:10.1097/MD.0000000000049131)
Supplement: Supplementary file 8 [file medi-105-e49131-s009.docx]

| Supplementary Table 9. Comparison of Gestational Abnormal ALT Risk (ATDP vs. NAT) by Modified Poisson Regression^a^ | | | | | |
| --- | --- | --- | --- | --- | --- |
| Variable | n (%) | Crude model | | Adjusted model | |
|  |  | RR (95%CI) | P | RR (95%CI) | P |
| ATDP | 22/99 (22.2) | Reference | Reference | Reference | Reference |
| NAP | 12/99 (12.1) | 0.55 (0.27, 1.10) | 0.091 | 0.51 (0.25, 1.05) | 0.067 |

ATDP, antiviral treatment during pregnancy; NAT, no antiviral treatment; RR, relative risk; CI, confidence interval; BMI, body mass index.

a Multivariate analyses were adjusted for maternal age, BMI, primigravida, primiparity by Poisson regression.
