# Supplementary material for: Maternal and infant growth outcomes following preconception antiviral therapy in chronic hepatitis B virus infection: A retrospective cohort study
Source: Medicine (Baltimore). 2026 Jun 12;105(24):e49131. doi: 10.1097/MD.0000000000049131 (PMC13268500; doi:10.1097/MD.0000000000049131)
Supplement: Supplementary file 9 [file medi-105-e49131-s010.docx]

| Supplementary Table 6. Pregnancy outcomes by Poisson regression after propensity score matching ^a^ | | | | | | | | | | | |
| --- | --- | --- | --- | --- | --- | --- | --- | --- | --- | --- | --- |
| Variables | ATBP N=99 | ATDP N=99 | Crude model ^b^ | | Adjusted model ^b^ | | NAT N=99 | Crude model ^c^ | | Adjusted model ^c^ | |
|  |  |  | RR (95%CI) | P | RR (95%CI) | P |  | RR (95%CI) | P | RR (95%CI) | P |
| Gestational abnormal ALT | 4 (4.0) | 22 (22.2) | 0.18 (0.07, 0.51) | 0.001 | 0.19 (0.07, 0.55) | 0.002 | 12(12.1) | 0.33 (0.11, 0.99) | 0.050 | 0.34 (0.12, 0.99) | 0.047 |
| HDP | 2 (2.0) | 4 (4.0) | 0.50 (0.09, 2,67) | 0.417 | 0.47 (0.09, 2.53) | 0.377 | 4(4.0) | 0.50 (0.09, 2.67) | 0.417 | 0.39 (0.07, 2.37) | 0.308 |
| GDM | 15 (15.2) | 10 (10.1) | 1.50 (0.71, 3.18) | 0.289 | 1.43 (0.69, 2.97) | 0.336 | 12(12.1) | 1.25 (0.62, 2.53) | 0.536 | 1.24 (0.62, 2.50) | 0.542 |
| Preterm birth | 2 (2.0) | 4 (4.0) | 0.50 (0.09, 2.67) | 0.417 | 0.71 (0.12, 4.24) | 0.705 | 4(4.0) | 0.50 (0.09, 2.67) | 0.417 | 0.51 (0.10, 2.61) | 0.420 |
| Postpartum hemorrhage | 30 (30.3) | 25 (25.3) | 1.20 (0.76, 1.89) | 0.429 | 1.18 (0.76, 1.84) | 0.462 | 33(33.3) | 0.91 (0.60, 1.37) | 0.647 | 0.91 (0.61, 1.35) | 0.626 |
| PROM | 14 (14.1) | 10 (10.1) | 1.40 (0.65, 3.00) | 0.387 | 1.24 (0.58, 2.69) | 0.579 | 14(14.1) | 1.0 (0.50, 1.99) | 1.000 | 1.02 (0.52, 2.01) | 0.951 |
| Abnormal amniotic fluid | 18 (18.2) | 14 (14.1) | 1.29 (0.68, 2.44) | 0.442 | 1.35 (0.72, 2.54) | 0.346 | 15(15.2) | 1.20 (0.64, 2.24) | 0.568 | 1.21 (0.65, 2.23) | 0.551 |
| ICP | 2 (2.0) | 5 (5.1) | 0.40 (0.08, 2.01) | 0.266 | 0.36 (0.07, 1.97) | 0.240 | 5(5.1) | 0.40 (0.08, 2.01) | 0.266 | 0.39 (0.07, 2.10) | 0.271 |

ATBP, antiviral treatment before pregnancy; ATDP, antiviral treatment during pregnancy; NAT, no antiviral treatment; RR, relative risk; CI, confidence interval; ALT, alanine aminotransferase; HDP, hypertensive disorders of pregnancy; GDM, gestational diabetes mellitus; PROM, premature rupture of the membranes; ICP, intrahepatic cholestasis of pregnancy; BMI, body mass index.

a Multivariate analyses were adjusted for maternal age, BMI, primigravida, primiparity by Poisson regression.

b ATBP vs. ATDP.c ATBP vs. NAT.
