# Supplementary material for: Maternal and infant growth outcomes following preconception antiviral therapy in chronic hepatitis B virus infection: A retrospective cohort study
Source: Medicine (Baltimore). 2026 Jun 12;105(24):e49131. doi: 10.1097/MD.0000000000049131 (PMC13268500; doi:10.1097/MD.0000000000049131)
Supplement: Supplementary file 10 [file medi-105-e49131-s011.docx]

| Supplementary Table 7. Growth parameters of children stratified by subgroup ^a^ | | | | | | | | | | | | |
| --- | --- | --- | --- | --- | --- | --- | --- | --- | --- | --- | --- | --- |
| Sex/Time points | Growth parameters,ln(Z-score-min+1) | ATBP N=99 | ATDP N=99 | Crude model ^b^ | | Adjusted model ^b^ | | NAT N=99 | Crude model ^c^ | | Adjusted model ^c^ | |
|  |  |  |  | E (95%CI) | P | E (95%CI) | P |  | E (95%CI) P | P | E (95%CI) | P |
| Boy, No. |  | 50 | 43 |  |  |  |  | 54 |  |  |  |  |
| 1 month | Weight | 1.9 ± 0.3 | 1.9 ± 0.1 | 0.05 (-0.04, 0.14) | 0.320 | 0.01 (-0.03, 0.05) | 0.685 | 1.9 ± 0.1 | 0.05 (-0.04, 0.14) | 0.261 | 0.01 (-0.04, 0.04) | 0.976 |
|  | Height | 1.9 ± 0.3 | 2.0 ± 0.2 | 0.04 (-0.06, 0.13) | 0.416 | 0.01 (-0.05, 0.05) | 0.917 | 2.0 ± 0.1 | 0.02 (-0.08, 0.11) | 0.741 | -0.04 (-0.08, 0.01) | 0.135 |
|  | Feeding method |  |  |  |  |  |  |  |  |  |  |  |
|  | Breast feeding | 18 (36.0) | 14 (32.6) | 1.11 (0.63, 1.95) | 0.728 | 1.13 (0.65, 1.98) | 0.657 | 13 (24.1) | 1.13 (0.95, 1.34) | 0.182 | 1.55 (0.86, 2.80) | 0.147 |
|  | Mixed feeding | 17 (34.0) | 19 (44.2) | 0.77 (0.46, 1.28) | 0.316 | 0.80 (0.48, 1.34) | 0.399 | 27 (50.0) | 0.85 (0.71, 1.03) | 0.094 | 0.70 (0.44, 1.12) | 0.139 |
|  | Artificial feeding | 15 (30.0) | 10 (23.3) | 1.29 (0.65, 2.57) | 0.469 | 1.22 (0.60, 2.47) | 0.583 | 14 (25.9) | 1.04 (0.88, 1.24) | 0.644 | 1.12 (0.60, 2.09) | 0.729 |
| 3 months | Weight | 1.5 ± 0.3 | 1.5 ± 0.2 | -0.01 (-0.10, 0.10) | 0.990 | -0.03 (-0.10,0.04) | 0.970 | 1.5 ± 0.2 | 0.05 (-0.02, 0.12) | 0.175 | -0.06 (-0.13, 0.01) | 0.061 |
|  | Height | 1.8 ± 0.3 | 1.8 ± 0.2 | 0.02 (-0.07, 0.12) | 0.614 | -0.01 (-0.07,0.05) | 0.734 | 1.8 ± 0.2 | 0.03 (-0.03, 0.10) | 0.284 | 0.01 (-0.07, 0.05) | 0.680 |
|  | Feeding method |  |  |  |  |  |  |  |  |  |  |  |
|  | Breast feeding | 21 (42.0) | 16 (37.2) | 1.13 (0.68, 1.87) | 0.640 | 1.16 (0.70, 1.92) | 0.571 | 20 (37.0) | 1.05 (0.87, 1.27) | 0.605 | 1.17 (0.73, 1.89) | 0.517 |
|  | Mixed feeding | 13 (26.0) | 11 (25.6) | 1.02 (0.51, 2.03) | 0.963 | 0.94 (0.46, 1.90) | 0.862 | 12 (22.2) | 1.04 (0.88, 1.22) | 0.653 | 1.08 (0.54, 2.18) | 0.831 |
|  | Artificial feeding | 16 (32.0) | 16 (37.2) | 0.86 (0.49, 1.51) | 0.598 | 0.90 (0.51, 1.58) | 0.715 | 22 (40.7) | 0.92 (0.76, 1.10) | 0.352 | 0.84 (0.50, 1.41) | 0.505 |
| 6 months | Weight | 1.6 ± 0.2 | 1.6 ± 0.2 | -0.01 (-0.08, 0.08) | 0.970 | -0.01 (-0.08, 0.07) | 0.924 | 1.6 ± 0.2 | 0.05 (-0.02, 0.11) | 0.157 | -0.04 (-0.10, 0.03) | 0.261 |
|  | Height | 1.3 ± 0.3 | 1.3 ± 0.2 | 0.02 (-0.09, 0.13) | 0.770 | -0.01 (-0.10, 0.08) | 0.832 | 1.3 ± 0.2 | 0.07 (-0.04, 0.17) | 0.209 | -0.01 (-0.10, 0.09) | 0.892 |
|  | Feeding method |  |  |  |  |  |  |  |  |  |  |  |
|  | Breast feeding | 9 (18.0) | 7 (16.3) | 1.11 (0.45, 2.72) | 0.827 | 1.15 (0.47, 2.82) | 0.764 | 9 (16.7) | 1.01 (0.88, 1.17) | 0.858 | 1.20 (0.54, 2.67) | 0.655 |
|  | Mixed feeding | 24 (48.0) | 20 (46.5) | 1.03 (0.67, 1.59) | 0.886 | 1.04 (0.67, 1.60) | 0.861 | 20 (37.0) | 1.12 (0.92, 1.35) | 0.256 | 1.32 (0.84, 2.09) | 0.232 |
|  | Artificial feeding | 17 (34.0) | 16 (37.2) | 0.91 (0.53, 1.58) | 0.747 | 0.89 (0.51, 1.55) | 0.685 | 25 (46.3) | 0.88 (0.73, 1.07) | 0.197 | 0.70 (0.43, 1.16) | 0.164 |
| 12months |  |  |  |  |  |  |  |  |  |  |  |  |
|  | Weight | 1.2 ± 0.3 | 1.2 ± 0.3 | 0.03 (-0.10, 0.15) | 0.698 | 0.03 (-0.09, 0.16) | 0.584 | 1.2 ± 0.2 | 0.02 (-0.09, 0.13) | 0.752 | 0.03 (-0.09, 0.14) | 0.654 |
|  | Height | 1.3 ± 0.3 | 1.3 ± 0.3 | 0.01 (-0.13, 0.13) | 0.995 | -0.01 (-0.13, 0.12) | 0.952 | 1.3 ± 0.3 | -0.01 (-0.12, 0.10) | 0.861 | -0.02 (-0.14, 0.09) | 0.713 |
|  | Teething | 7.2 ± 2.2 | 6.9 ± 1.5 | -0.27 (-1.01, 0.46) | 0.463 | -0.33 (-1.06, 0.41) | 0.382 | 7.1 ± 2.5 | -0.06 (-0.95, 0.84) | 0.901 | -0.16 (-1.05,0.74) | 0.730 |
|  | Fontanelle closure | 18 (36.0) | 15 (34.9) | 0.97 (0.56, 1.68) | 0.911 | 0.93 (0.53, 1.60) | 0.780 | 22 (40.7) | 1.13 (0.69, 1.85) | 0.621 | 1.04 (0.63,1.70) | 0.887 |
| Girl, No. |  | 49 | 56 |  |  |  |  | 45 |  |  |  |  |
| 1 month | Weight | 1.9 ± 0.2 | 1.9 ± 0.1 | -0.01 (-0.05, 0.05) | 1.000 | -0.01 (-0.05, 0.03) | 0.726 | 1.9 ± 0.1 | 0.01 (-0.05, 0.06) | 0.940 | -0.01 (-0.06,0.04) | 0.780 |
|  | Height | 2.0 ± 0.2 | 2.0 ± 0.1 | 0.01 (-0.05, 0.05), | 0.943 | -0.01 (-0.05, 0.03) | 0.724 | 2.0 ± 0.1 | 0.01 (-0.05, 0.07) | 0.688 | 0.01 (-0.05,0.06) | 0.904 |
|  | Feeding method |  |  |  |  |  |  |  |  |  |  |  |
|  | Breast feeding | 21 (42.9) | 23 (41.1) | 1.04 (0.67, 1.64) | 0.853 | 1.01 (0.64, 1.60) | 0.959 | 20 (44.4) | 0.96 (0.61, 1.53) | 0.877 | 0.99 (0.63, 1.55) | 0.951 |
|  | Mixed feeding | 16 (32.7) | 18 (32.1) | 1.02 (0.58, 1.77) | 0.956 | 1.03 (0.60, 1.78) | 0.926 | 15 (33.3) | 0.98 (0.55, 1.74) | 0.944 | 0.95 (0.54, 1.69) | 0.871 |
|  | Artificial feeding | 12 (24.5) | 15 (26.8) | 0.91 (0.48, 1.76) | 0.789 | 0.93 (0.48, 1.78) | 0.926 | 10 (22.2) | 1.10 (0.53, 2.30) | 0.796 | 1.08 (0.51, 2.28) | 0.840 |
| 3 months | Weight | 1.5 ± 0.2 | 1.5 ± 0.2 | 0.05 (-0.02, 0.12) | 0.175 | 0.04 (-0.02, 0.11) | 0.197 | 1.5 ± 0.2 | 0.02 (-0.06, 0.10) | 0.640 | 0.01 (-0.07, 0.09) | 0.799 |
|  | Height | 1.7 ± 0.2 | 1.8 ± 0.1 | 0.03 (-0.03, 0.10) | 0.284 | 0.03 (-0.03, 0.08) | 0.385 | 1.8 ± 0.2 | 0.05 (-0.02, 0.11) | 0.158 | 0.04 (-0.02,0.11) | 0.196 |
|  | Feeding method |  |  |  |  |  |  |  |  |  |  |  |
|  | Breast feeding | 22 (44.9) | 27 (48.2) | 0.93 (0.62, 1.41) | 0.735 | 0.93 (0.61, 1.41) | 0.729 | 17 (37.8) | 1.19 (0.73, 1.93) | 0.487 | 1.22 (0.75, 1.97) | 0.424 |
|  | Mixed feeding | 15 (30.6) | 13 (23.2) | 1.32 (0.70, 2.49) | 0.394 | 1.33 (0.70, 2.53) | 0.380 | 15 (33.3) | 0.92 (0.51, 1.66) | 0.777 | 0.89 (0.49, 1.62) | 0.708 |
|  | Artificial feeding | 12 (24.5) | 16 (28.6) | 0.86 (0.45, 1.63) | 0.638 | 0.85 (0.45, 1.61) | 0.613 | 13 (28.9) | 0.85 (0.43, 1.66) | 0.630 | 0.83 (0.42, 1.64) | 0.600 |
| 6 months | Weight | 1.6 ± 0.2 | 1.6 ± 0.2 | 0.05 (-0.02, 0.11) | 0.157 | 0.04 (-0.02, 0.10) | 0.187 | 1.6 ± 0.3 | -0.02 (-0.12, 0.08) | 0.692 | -0.03 (-0.13, 0.08) | 0.625 |
|  | Height | 1.3 ± 0.3 | 1.3 ± 0.2 | 0.07 (-0.04, 0.17) | 0.209 | 0.05 (-0.04, 0.14) | 0.266 | 1.4 ± 0.2 | -0.08 (-0.03, 0.18) | 0.149 | 0.07 (-0.03, 0.17) | 0.176 |
|  | Feeding method |  |  |  |  |  |  |  |  |  |  |  |
|  | Breast feeding | 10 (20.4) | 17 (30.4) | 0.67 (0.34, 1.33) | 0.253 | 0.63 (0.32, 1.24) | 0.178 | 9 (20.0) | 1.02 (0.46, 2.28) | 0.961 | 1.04 (0.47, 2.35) | 0.917 |
|  | Mixed feeding | 20 (40.8) | 19 (33.9) | 1.20 (0.73, 1.98) | 0.466 | 1.23 (0.75, 2.02) | 0.412 | 16 (35.6) | 1.15 (0.68, 1.93) | 0.602 | 1.16 (0.69, 1.96) | 0.570 |
|  | Artificial feeding | 19 (38.8) | 20 (35.7) | 1.09 (0.66, 1.79) | 0.746 | 1.09 (0.66, 1.79) | 0.729 | 20 (44.4) | 0.87 (0.54, 1.41) | 0.577 | 0.86 (0.53, 1.39) | 0.545 |
| 12months | Weight | 1.2 ± 0.3 | 1.2 ± 0.2 | -0.05 (-0.15, 0.05) | 0.325 | -0.04 (-0.12, 0.05) | 0.408 | 1.2 ± 0.2 | 0.06 (-0.05, 0.17) | 0.295 | 0.05 (-0.06, 0.17) | 0.344 |
|  | Height | 1.3 ± 0.3 | 1.3 ± 0.2 | -0.03 (-0.13, 0.06) | 0.504 | -0.02 (-0.11, 0.07) | 0.674 | 1.3 ± 0.2 | 0.04 (-0.05, 0.14) | 0.369 | 0.04 (-0.05, 0.13) | 0.412 |
|  | Teething | 6.5 ± 2.2 | 6.6 ± 2.0 | -0.04 (-0.84, 0.77) | 0.932 | 0.02 (-0.79, 0.82) | 0.970 | 7.1 ± 1.8 | 0.62 (-0.19, 1.43) | 0.135 | 0.59 (-0.21, 1.40) | 0.148 |
|  | Fontanelle closure | 22 (44.9) | 16 (28.6) | 1.57 (0.94, 2.64) | 0.087 | 1.61 (0.96, 2.70) | 0.070 | 16 (35.6) | 0.79 (0.48, 1.31) | 0.361 | 0.81(0.49,1.34), | 0.406 |

ATBP, antiviral treatment before pregnancy; ATDP, antiviral treatment during pregnancy; NAT, no antiviral treatment; E, estimate; CI, confidence interval.

a Multivariate analyses were adjusted for weight (birth), height (birth) and feeding method at 1, 3 and 6 months and adjusted for weight (birth), height (birth) at 12 months. Weight and height were transformed to Z-scores of weight-for-age and height-for-age, using Ln (Z score-min+1) for analysis.

b ATBP vs. ATDP c ATBP vs. NAT
