# Supplementary material for: Maternal and infant growth outcomes following preconception antiviral therapy in chronic hepatitis B virus infection: A retrospective cohort study
Source: Medicine (Baltimore). 2026 Jun 12;105(24):e49131. doi: 10.1097/MD.0000000000049131 (PMC13268500; doi:10.1097/MD.0000000000049131)
Supplement: Supplementary file 11 [file medi-105-e49131-s012.docx]

| Supplementary Table 11. Pregnancy outcomes by Poisson regression after propensity score matching ^a^ | | | | | | | | | | | |
| --- | --- | --- | --- | --- | --- | --- | --- | --- | --- | --- | --- |
| Variables | ATBP N=99 | ATDP N=99 | Crude model ^b^ | | Adjusted model ^b^ | | NAT N=99 | Crude model ^c^ | | Adjusted model ^c^ | |
|  |  |  | RR (95%CI) | P | RR (95%CI) | P |  | RR (95%CI) | P | RR (95%CI) | P |
| Gestational abnormal ALT | 4 (4.0) | 22 (22.2) | 0.182 (0.063, 0.528) | 0.002 | 0.169 (0.057, 0.499) | 0.001 | 12 (12.1) | 0.333 (0.108, 1.034) | 0.057 | 0.317 (0.102, 0.987) | 0.047 |
| HDP | 2 (2.0) | 4 (4.0) | 0.500 (0.092, 2.730) | 0.423 | 0.519 (0.089, 3.023) | 0.466 | 4 (4.0) | 0.500 (0.092, 2.730) | 0.423 | 0.471 (0.085, 2.613) | 0.389 |
| GDM | 15 (15.2) | 10 (10.1) | 1.500 (0.674, 3.339) | 0.321 | 1.209 (0.530, 2.759) | 0.651 | 12 (12.1) | 1.250 (0.585, 2.670) | 0.565 | 1.130 (0.524, 2.437) | 0.755 |
| Preterm birth | 2 (2.0) | 4 (4.0) | 0.500 (0.092, 2.730) | 0.423 | 0.839 (0.133, 5.277) | 0.851 | 4 (4.0) | 0.500 (0.092, 2.730) | 0.423 | 0.500 (0.090, 2.769) | 0.428 |
| Postpartum hemorrhage | 30 (30.3) | 25 (25.3) | 1.200 (0.706, 2.040) | 0.501 | 1.037 (0.600, 1.792) | 0.896 | 33 (33.3) | 0.909 (0.554, 1.491) | 0.706 | 0.889 (0.541, 1.460) | 0.642 |
| PROM | 14 (14.1) | 10 (10.1) | 1.400 (0.622, 3.152) | 0.416 | 1.266 (0.546, 2.932) | 0.582 | 13 (13.1) | 1.077 (0.506, 2.291) | 0.847 | 1.033 (0.483, 2.212) | 0.932 |
| Abnormal amniotic fluid | 18 (18.2) | 14 (14.1) | 1.286 (0.639, 2.585) | 0.481 | 1.293 (0.627, 2.668) | 0.487 | 15 (15.2) | 1.200 (0.605, 2.381) | 0.602 | 1.164 (0.585, 2.314) | 0.665 |
| ICP | 2 (2.0) | 5 (5.1) | 0.400 (0.078, 2.062) | 0.273 | 0.348 (0.062, 1.947) | 0.229 | 5 (5.1) | 0.400 (0.078, 2.062) | 0.273 | 0.433 (0.083, 2.263) | 0.321 |

ATBP, antiviral treatment before pregnancy; ATDP, antiviral treatment during pregnancy; NAT, no antiviral treatment; RR, relative risk; CI, confidence interval; ALT, alanine aminotransferase; HDP, hypertensive disorders of pregnancy; GDM, gestational diabetes mellitus; PROM, premature rupture of the membranes; ICP, intrahepatic cholestasis of pregnancy; BMI, body mass index.

a Multivariate analyses were adjusted for maternal age, BMI, primigravida, primiparity by Poisson regression.

b ATBP vs. ATDP. c ATBP vs. NAT.
