# Supplementary material for: Maternal and infant growth outcomes following preconception antiviral therapy in chronic hepatitis B virus infection: A retrospective cohort study
Source: Medicine (Baltimore). 2026 Jun 12;105(24):e49131. doi: 10.1097/MD.0000000000049131 (PMC13268500; doi:10.1097/MD.0000000000049131)
Supplement: Supplementary file 12 [file medi-105-e49131-s013.docx]

| Supplementary Table 12. Growth parameters of children analyzed by linear regression with multiple variables ^a^ | | | | | |
| --- | --- | --- | --- | --- | --- |
| Variables | ATBP | ATDP | P ^b^ | NAT | P ^c^ |
| Children | 99 | 99 |  | 99 |  |
| 1 month |  |  |  |  |  |
| Weight | 1.89±0.24 | 1.92±0.13 | 0.217 | 1.92±0.12 | 0.188 |
| Height | 1.96±0.15 | 1.96±0.11 | 0.748 | 1.95±0.12 | 0.325 |
| 3 months |  |  |  |  |  |
| Weight | 1.48±0.26 | 1.50±0.17 | 0.459 | 1.48±0.18 | 0.975 |
| Height | 1.74±0.25 | 1.77±0.15 | 0.320 | 1.79±0.16 | 0.122 |
| 6 months |  |  |  |  |  |
| Weight | 1.62±0.18 | 1.64±0.18 | 0.490 | 1.59±0.23 | 0.338 |
| Height | 1.29±0.31 | 1.32±0.24 | 0.472 | 1.34±0.23 | 0.291 |
| 12months |  |  |  |  |  |
|  | 1.17±0.33 | 1.21±0.24 | 0.431 | 1.20±0.22 | 0.482 |
|  | 1.30±0.30 | 1.32±0.27 | 0.656 | 1.32±0.23 | 0.666 |
| Teething | 7.35±2.70 | 6.87±1.63 | 0.128 | 7.69±3.35 | 0.442 |
| Fontanelle closure, no. | 40 (44.4) | 31 (31.3) | 0.125 | 38 (38.4) | 0.498 |

ATBP, antiviral treatment before pregnancy; ATDP, antiviral treatment during pregnancy; NAT, no antiviral treatment.

a Multivariate analyses were adjusted for weight (birth), height (birth) and feeding method at 1, 3 and 6 months and adjusted for weight (birth), height (birth) at 12 months. Weight and height were transformed to Z-scores of weight-for-age and height-for-age, using Ln (Z score-min+1) for analysis.

b ATBP vs. ATDP

c ATBP vs. NAT
